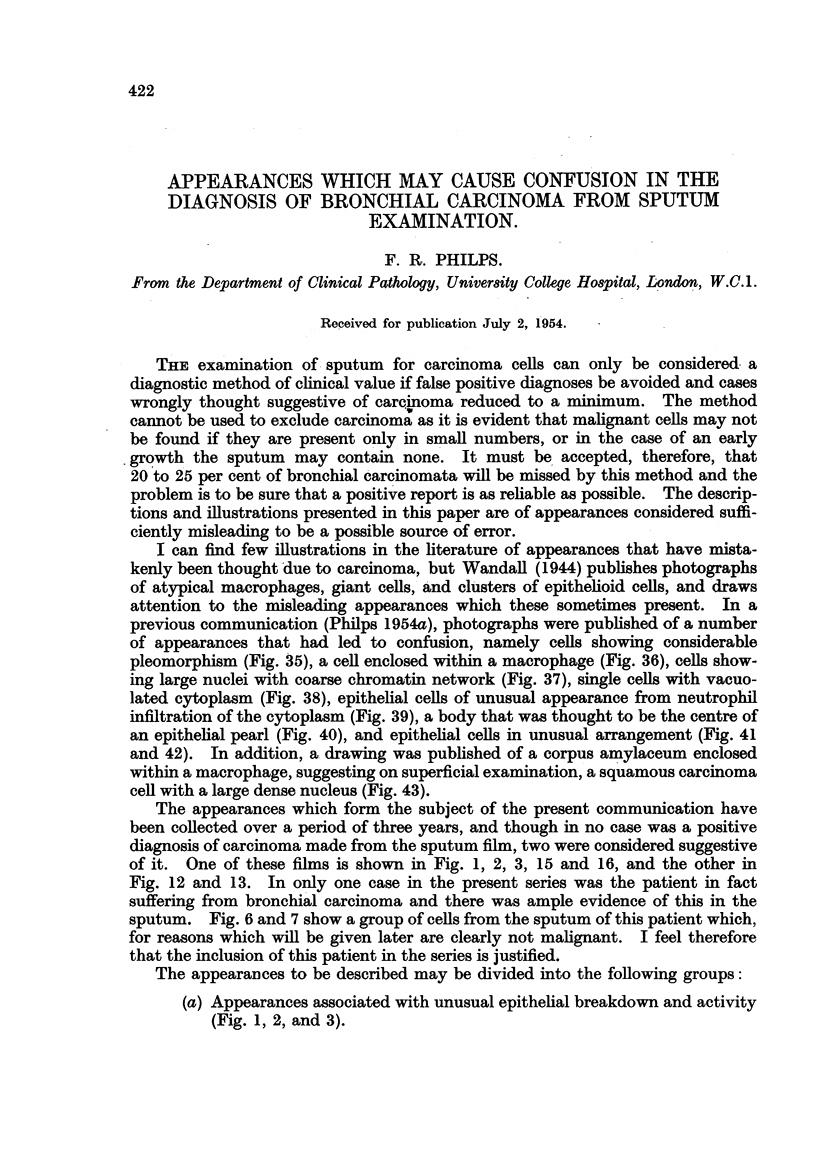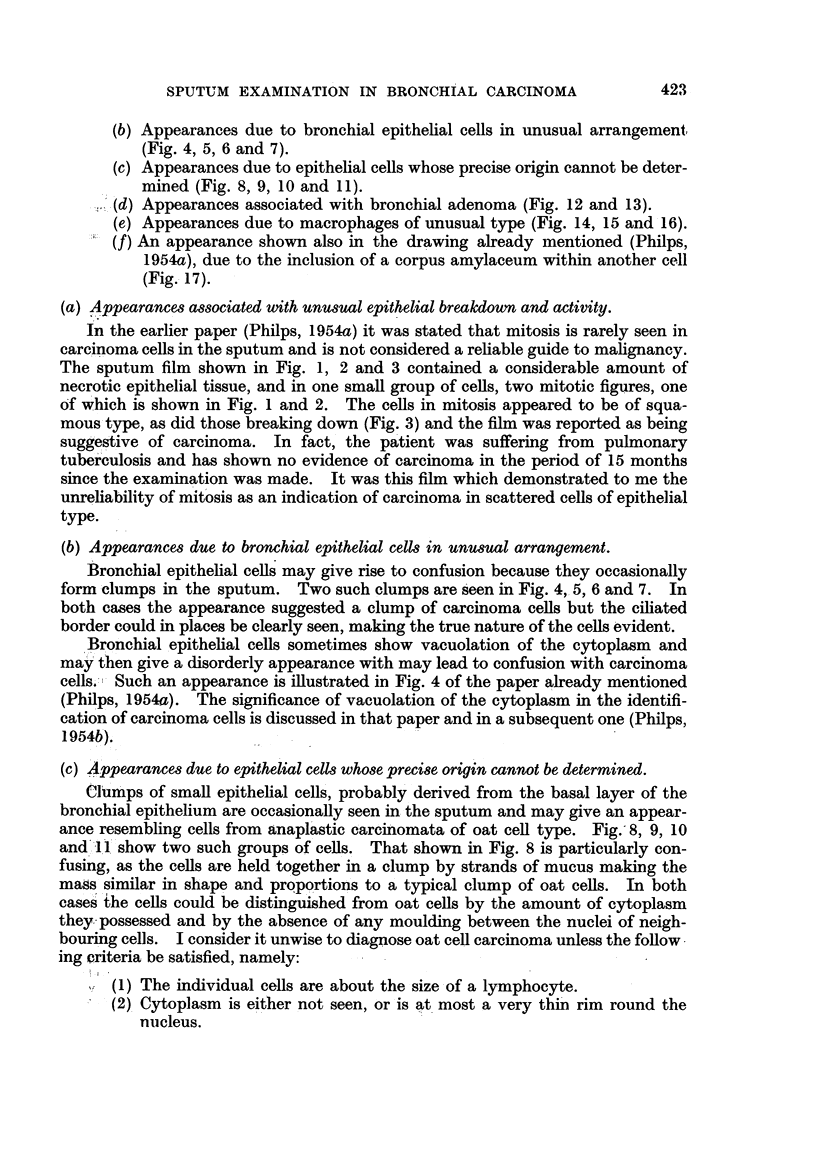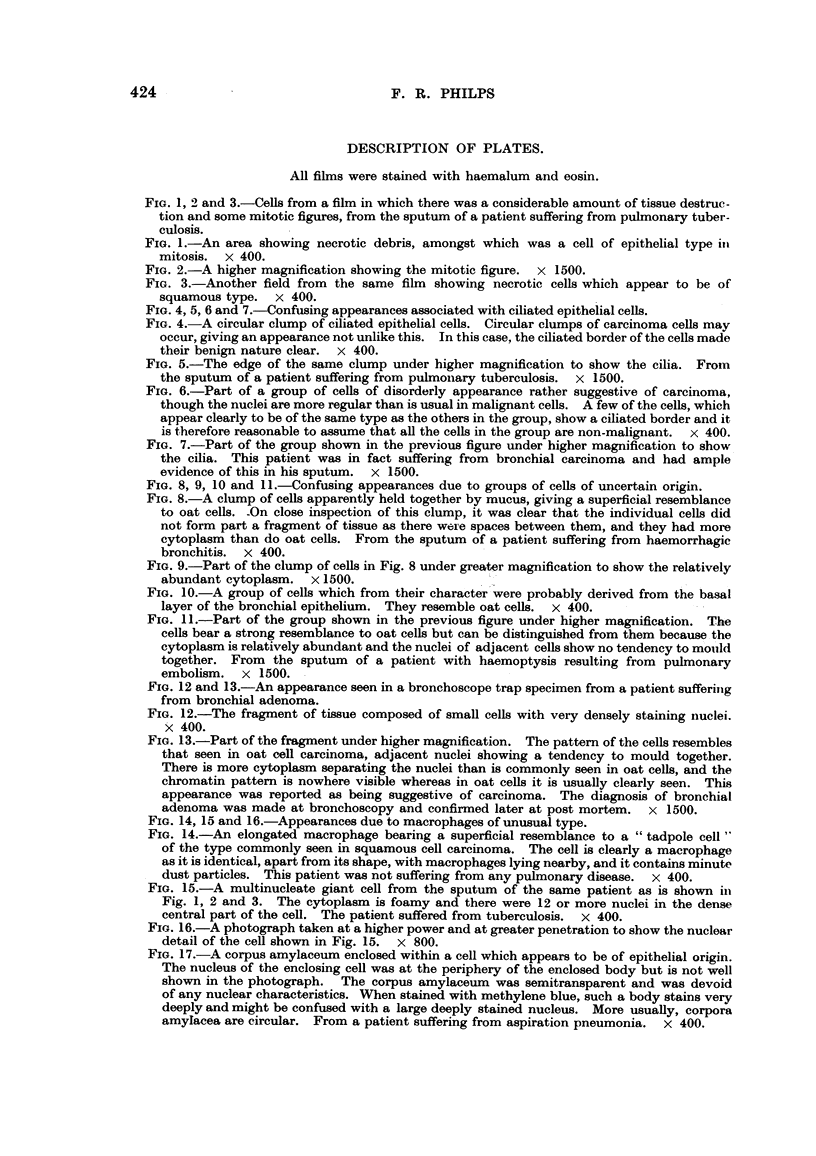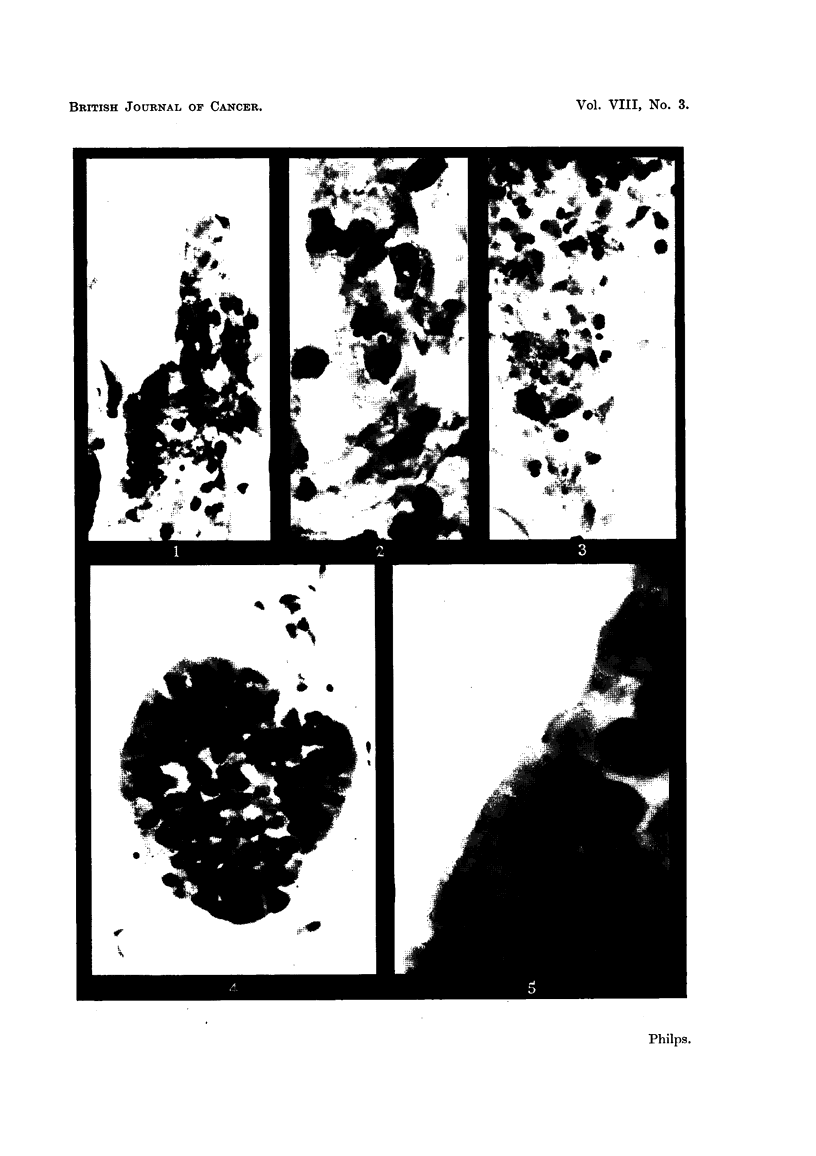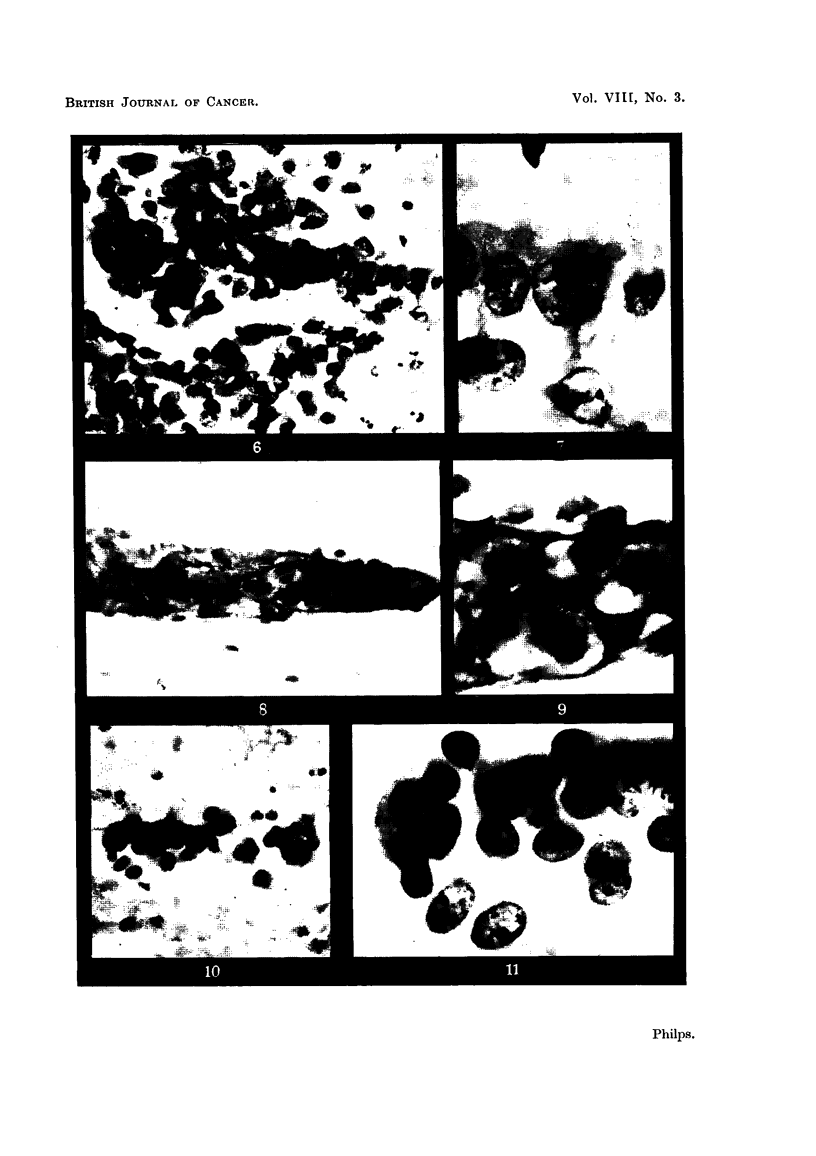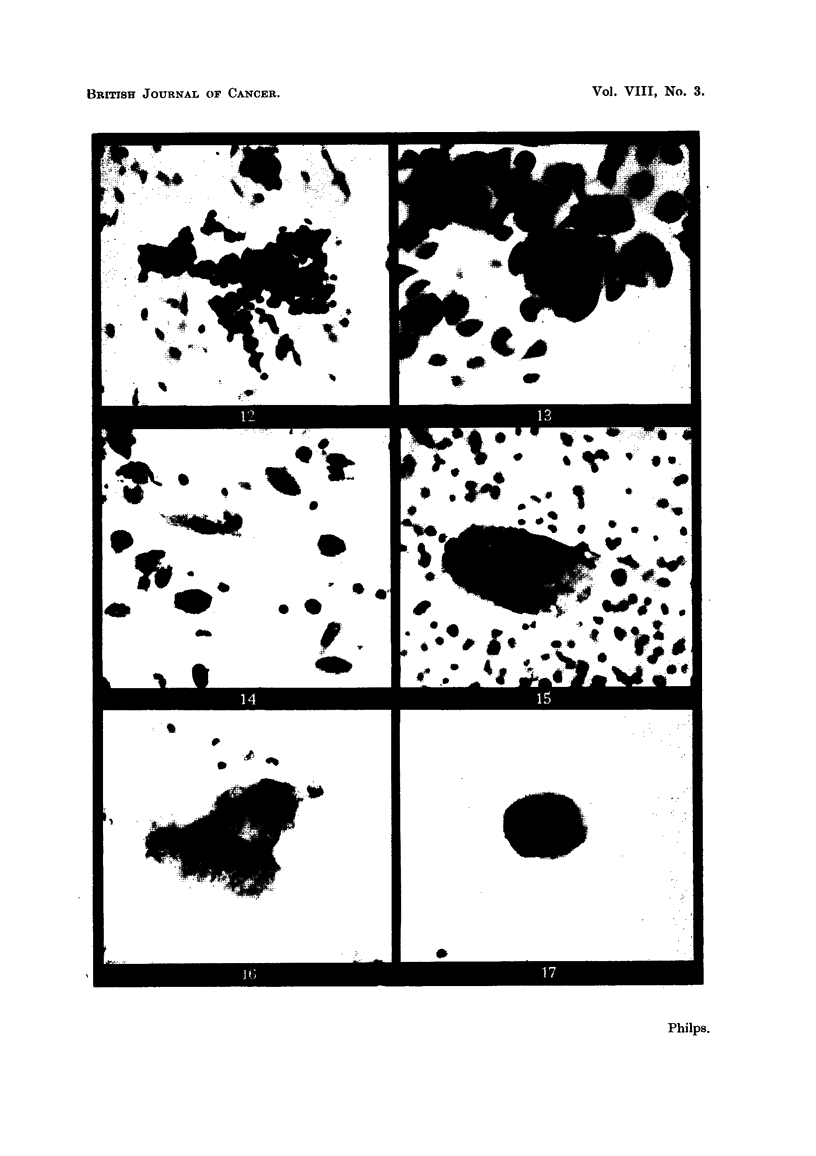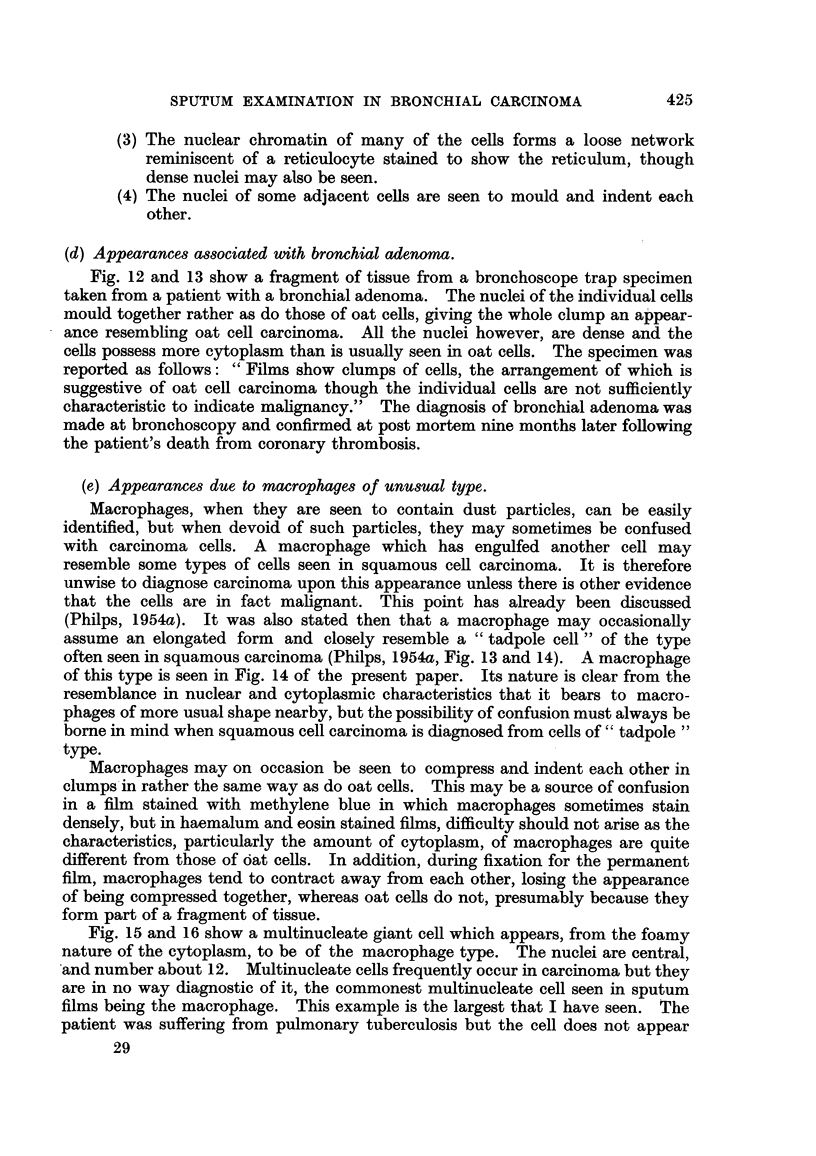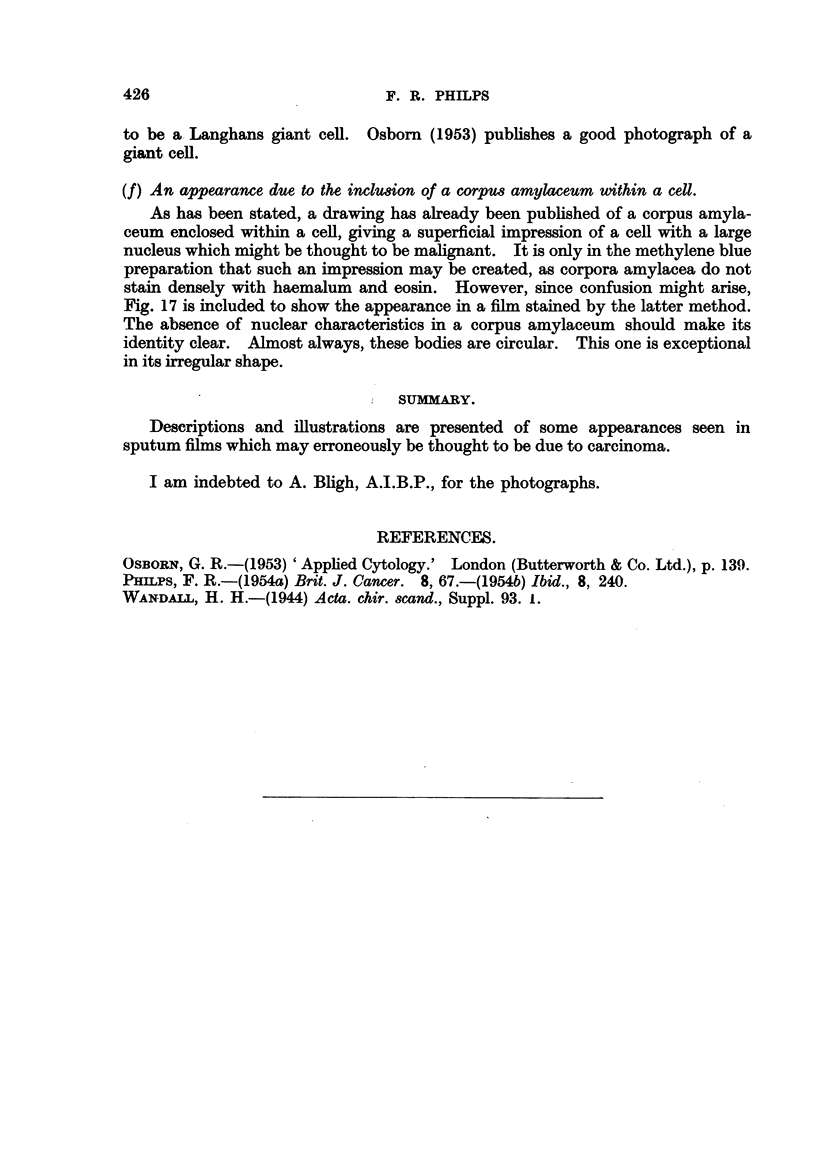# Appearances which may Cause Confusion in the Diagnosis of Bronchial Carcinoma from Sputum Examination

**DOI:** 10.1038/bjc.1954.44

**Published:** 1954-09

**Authors:** F. R. Philps

## Abstract

**Images:**


### APPEARANCES WHICH MAY CAUSE CONFUSION IN THE DIAGNOSIS OF BRONCHIAL CARCINOMA FROM SPUTUM EXAMINATION.

F. R. PHILPS.

*From the Department of Clinical Pathology, University College Hospital, London, W.C.*1.

Received for publication July 2, 1954.

THE examination of sputum for carcinoma cells can only be considered a diagnostic method of clinical value if false positive diagnoses be avoided and cases wrongly thought suggestive of carcinoma reduced to a minimum. The method cannot be used to exclude carcinoma as it is evident that malignant cells may not be found if they are present only in small numbers, or in the case of an early growth the sputum may contain none. It must be accepted, therefore, that 20 to 25 per cent of bronchial carcinomata will be missed by this method and the problem is to be sure that a positive report is as reliable as possible. The descriptions and illustrations presented in this paper are of appearances considered sufficiently misleading to be a possible source of error.

I can find few illustrations in the literature of appearances that have mistakenly been thought due to carcinoma, but Wandall (1944) publishes photographs of atypical macrophages, giant cells, and clusters of epithelioid cells, and draws attention to the misleading appearances which these sometimes present. In a previous communication (Philps 1954*a*), photographs were published of a number of appearances that had led to confusion, namely cells showing considerable pleomorphism (Fig. 35), a cell enclosed within a macrophage (Fig. 36), cells showing large nuclei with coarse chromatin network (Fig. 37), single cells with vacuolated cytoplasm (Fig. 38), epithelial cells of unusual appearance from neutrophil infiltration of the cytoplasm (Fig. 39), a body that was thought to be the centre of an epithelial pearl (Fig. 40), and epithelial cells in unusual arrangement (Fig. 41 and 42). In addition, a drawing was published of a corpus amylaceum enclosed within a macrophage, suggesting on superficial examination, a squamous carcinoma cell with a large dense nucleus (Fig. 43).

The appearances which form the subject of the present communication have been collected over a period of three years, and though in no case was a positive diagnosis of carcinoma made from the sputum film, two were considered suggestive of it. One of these films is shown in Fig. 1, 2, 3, 15 and 16, and the other in Fig. 12 and 13. In only one case in the present series was the patient in fact suffering from bronchial carcinoma and there was ample evidence of this in the sputum. Fig. 6 and 7 show a group of cells from the sputum of this patient which, for reasons which will be given later are clearly not malignant. I feel therefore that the inclusion of this patient in the series is justified.

The appearances to be described may be divided into the following groups:

(*a*) Appearances associated with unusual epithelial breakdown and activity (Fig. 1, 2, and 3).

(*b*) Appearances due to bronchial epithelial cells in unusual arrangement (Fig. 4, 5, 6 and 7).
(*c*) Appearances due to epithelial cells whose precise origin cannot be determined (Fig. 8, 9, 10 and 11).
(*d*) Appearances associated with bronchial adenoma (Fig. 12 and 13).
(*e*) Appearances due to macrophages of unusual type (Fig. 14, 15 and 16).
(*f*) An appearance shown also in the drawing already mentioned (Philps, 1954*a*), due to the inclusion of a corpus amylaceum within another cell (Fig. 17).

(*a*) *Appearances associated with unusual epithelial breakdown and activity.*

In the earlier paper (Philps, 1954*a*) it was stated that mitosis is rarely seen in carcinoma cells in the sputum and is not considered a reliable guide to malignancy. The sputum film shown in Fig. 1, 2 and 3 contained a considerable amount of necrotic epithelial tissue, and in one small group of cells, two mitotic figures, one of which is shown in Fig. 1 and 2. The cells in mitosis appeared to be of squamous type, as did those breaking down (Fig. 3) and the film was reported as being suggestive of carcinoma. In fact, the patient was suffering from pulmonary tuberculosis and has shown no evidence of carcinoma in the period of 15 months since the examination was made. It was this film which demonstrated to me the unreliability of mitosis as an indication of carcinoma in scattered cells of epithelial type.

(*b*) *Appearances due to bronchial epithelial cells in unusual arrangement.*

Bronchial epithelial cells may give rise to confusion because they occasionally form clumps in the sputum. Two such clumps are seen in Fig. 4, 5, 6 and 7. In both cases the appearance suggested a clump of carcinoma cells but the ciliated border could in places be clearly seen, making the true nature of the cells evident.

Bronchial epithelial cells sometimes show vacuolation of the cytoplasm and may then give a disorderly appearance with may lead to confusion with carcinoma cells. Such an appearance is illustrated in Fig. 4 of the paper already mentioned (Philps, 1954*a*). The significance of vacuolation of the cytoplasm in the identification of carcinoma cells is discussed in that paper and in a subsequent one (Philps, 1954*b*).

(*c*) *Appearances due to epithelial cells whose precise origin cannot be determined.*

Clumps of small epithelial cells, probably derived from the basal layer of the bronchial epithelium are occasionally seen in the sputum and may give an appearance resembling cells from anaplastic carcinomata of oat cell type. Fig. 8, 9, 10 and 11 show two such groups of cells. That shown in Fig. 8 is particularly confusing, as the cells are held together in a clump by strands of mucus making the mass similar in shape and proportions to a typical clump of oat cells. In both cases the cells could be distinguished from oat cells by the amount of cytoplasm they possessed and by the absence of any moulding between the nuclei of neighbouring cells. I consider it unwise to diagnose oat cell carcinoma unless the following criteria be satisfied, namely:

(1) The individual cells are about the size of a lymphocyte.
(2) Cytoplasm is either not seen, or is at most a very thin rim round the nucleus.

### DESCRIPTION OF PLATES.

All films were stained with haemalum and eosin.

FIG. 1, 2 and 3.—Cells from a film in which there was a considerable amount of tissue destruction and some mitotic figures, from the sputum of a patient suffering from pulmonary tuberculosis.

FIG. 1.—An area showing necrotic debris, amongst which was a cell of epithelial type in mitosis. × 400.

FIG. 2.—A higher magnification showing the mitotic figure. × 1500.

FIG. 3.—Another field from the same film showing necrotic cells which appear to be of squamous type. × 400.

FIG. 4, 5, 6 and 7.—Confusing appearances associated with ciliated epithelial cells.

FIG. 4.—A circular clump of ciliated epithelial cells. Circular clumps of carcinoma cells may occur, giving an appearance not unlike this. In this case, the ciliated border of the cells made their benign nature clear. × 400.

FIG. 5.—The edge of the same clump under higher magnification to show the cilia. From the sputum of a patient suffering from pulmonary tuberculosis. × 1500.

FIG. 6.—Part of a group of cells of disorderly appearance rather suggestive of carcinoma, though the nuclei are more regular than is usual in malignant cells. A few of the cells, which appear clearly to be of the same type as the others in the group, show a ciliated border and it is therefore reasonable to assume that all the cells in the group are non-malignant. × 400.

FIG. 7.—Part of the group shown in the previous figure under higher magnification to show the cilia. This patient was in fact suffering from bronchial carcinoma and had ample evidence of this in his sputum. × 1500.

FIG. 8, 9, 10 and 11.—Confusing appearances due to groups of cells of uncertain origin.

FIG. 8.—A clump of cells apparently held together by mucus, giving a superficial resemblance to oat cells. On close inspection of this clump, it was clear that the individual cells did not form part a fragment of tissue as there were spaces between them, and they had more cytoplasm than do oat cells. From the sputum of a patient suffering from haemorrhagic bronchitis. × 400.

FIG. 9.—Part of the clump of cells in Fig. 8 under greater magnification to show the relatively abundant cytoplasm. ×1500.

FIG. 10.—A group of cells which from their character were probably derived from the basal layer of the bronchial epithelium. They resemble oat cells. × 400.

FIG. 11.—Part of the group shown in the previous figure under higher magnification. The cells bear a strong resemblance to oat cells but can be distinguished from them because the cytoplasm is relatively abundant and the nuclei of adjacent cells show no tendency to mould together. From the sputum of a patient with haemoptysis resulting from pulmonary embolism. × 1500.

FIG. 12 and 13.—An appearance seen in a bronchoscope trap specimen from a patient suffering from bronchial adenoma.

FIG. 12.—The fragment of tissue composed of small cells with very densely staining nuclei. × 400.

FIG. 13.—Part of the fragment under higher magnification. The pattern of the cells resembles that seen in oat cell carcinoma, adjacent nuclei showing a tendency to mould together. There is more cytoplasm separating the nuclei than is commonly seen in oat cells, and the chromatin pattern is nowhere visible whereas in oat cells it is usually clearly seen. This appearance was reported as being suggestive of carcinoma. The diagnosis of bronchial adenoma was made at bronchoscopy and confirmed later at post mortem. × 1500.

FIG. 14, 15 and 16.—Appearances due to macrophages of unusual type.

FIG. 14.—An elongated macrophage bearing a superficial resemblance to a "tadpole cell" of the type commonly seen in squamous cell carcinoma. The cell is clearly a macrophage as it is identical, apart from its shape, with macrophages lying nearby, and it contains minute dust particles. This patient was not suffering from any pulmonary disease. × 400.

FIG. 15.—A multinucleate giant cell from the sputum of the same patient as is shown in Fig. 1, 2 and 3. The cytoplasm is foamy and there were 12 or more nuclei in the dense central part of the cell. The patient suffered from tuberculosis. × 400.

FIG. 16.—A photograph taken at a higher power and at greater penetration to show the nuclear detail of the cell shown in Fig. 15. × 800.

FIG. 17.—A corpus amylaceum enclosed within a cell which appears to be of epithelial origin. The nucleus of the enclosing cell was at the periphery of the enclosed body but is not well shown in the photograph. The corpus amylaceum was semitransparent and was devoid of any nuclear characteristics. When stained with methylene blue, such a body stains very deeply and might be confused with a large deeply stained nucleus. More usually, corpora amylacea are circular. From a patient suffering from aspiration pneumonia. × 400.

Philps.

6 7 8 9 10 11

Philps.

12

13

14

15

16

17

Philps.

(3) The nuclear chromatin of many of the cells forms a loose network reminiscent of a reticulocyte stained to show the reticulum, though dense nuclei may also be seen.
(4) The nuclei of some adjacent cells are seen to mould and indent each other.

(*d*) *Appearances associated with bronchial adenoma.*

Fig. 12 and 13 show a fragment of tissue from a bronchoscope trap specimen taken from a patient with a bronchial adenoma. The nuclei of the individual cells mould together rather as do those of oat cells, giving the whole clump an appearance resembling oat cell carcinoma. All the nuclei however, are dense and the cells possess more cytoplasm than is usually seen in oat cells. The specimen was reported as follows: " Films show clumps of cells, the arrangement of which is suggestive of oat cell carcinoma though the individual cells are not sufficiently characteristic to indicate malignancy." The diagnosis of bronchial adenoma was made at bronchoscopy and confirmed at post mortem nine months later following the patient's death from coronary thrombosis.

(*e*) *Appearances due to macrophages of unusual type.*

Macrophages, when they are seen to contain dust particles, can be easily identified, but when devoid of such particles, they may sometimes be confused with carcinoma cells. A macrophage which has engulfed another cell may resemble some types of cells seen in squamous cell carcinoma. It is therefore unwise to diagnose carcinoma upon this appearance unless there is other evidence that the cells are in fact malignant. This point has already been discussed (Philps, 1954*a*). It was also stated then that a macrophage may occasionally assume an elongated form and closely resemble a " tadpole cell " of the type often seen in squamous carcinoma (Philps, 1954*a*, Fig. 13 and 14). A macrophage of this type is seen in Fig. 14 of the present paper. Its nature is clear from the resemblance in nuclear and cytoplasmic characteristics that it bears to macrophages of more usual shape nearby, but the possibility of confusion must always be borne in mind when squamous cell carcinoma is diagnosed from cells of " tadpole " type.

Macrophages may on occasion be seen to compress and indent each other in clumps in rather the same way as do oat cells. This may be a source of confusion in a film stained with methylene blue in which macrophages sometimes stain densely, but in haemalum and eosin stained films, difficulty should not arise as the characteristics, particularly the amount of cytoplasm, of macrophages are quite different from those of oat cells. In addition, during fixation for the permanent film, macrophages tend to contract away from each other, losing the appearance of being compressed together, whereas oat cells do not, presumably because they form part of a fragment of tissue.

Fig. 15 and 16 show a multinucleate giant cell which appears, from the foamy nature of the cytoplasm, to be of the macrophage type. The nuclei are central, and number about 12. Multinucleate cells frequently occur in carcinoma but they are in no way diagnostic of it, the commonest multinucleate cell seen in sputum films being the macrophage. This example is the largest that I have seen. The patient was suffering from pulmonary tuberculosis but the cell does not appear to be a Langhans giant cell. Osborn (1953) publishes a good photograph of a giant cell.

(*f*) *An appearance due to the inclusion of a corpus amylaceum within a cell.*

As has been stated, a drawing has already been published of a corpus amylaceum enclosed within a cell, giving a superficial impression of a cell with a large nucleus which might be thought to be malignant. It is only in the methylene blue preparation that such an impression may be created, as corpora amylacea do not stain densely with haemalum and eosin. However, since confusion might arise, Fig. 17 is included to show the appearance in a film stained by the latter method. The absence of nuclear characteristics in a corpus amylaceum should make its identity clear. Almost always, these bodies are circular. This one is exceptional in its irregular shape.

### SUMMARY.

Descriptions and illustrations are presented of some appearances seen in sputum films which may erroneously be thought to be due to carcinoma.

I am indebted to A. Bligh, A.I.B.P., for the photographs.

### REFERENCES.

OSBORN, G. R.—(1953) ‘ Applied Cytology.’ London (Butterworth & Co. Ltd.), p. 139.
PHILPS, F. R.—(1954*a*) *Brit. J. Cancer.* 8, 67.—(1954*b*) *Ibid.*, 8, 240.
WANDALL, H. H.—(1944) *Acta. chir. scand.*, Suppl. 93. 1.